# Comparison of Performance on the Clock Drawing Test Using Three Different Scales in Dialysis Patients

**DOI:** 10.1155/2020/7963837

**Published:** 2020-09-22

**Authors:** Taim Abdullah Muayqil, Ahmad Raed Tarakji, Abdullah Mohammad Khattab, Nasser Talal Balbaid, Ahmad Mohedeen Al-Dawalibi, Sami Ahmed Alqarni, Reema Ali Hazazi, Mohammed H. Alanazy

**Affiliations:** ^1^Neurology Unit, Department of Internal Medicine, College of Medicine, King Saud University, PO Box 7805 (38) Riyadh 11472, Saudi Arabia; ^2^Nephrology Unit, Department of Internal Medicine, College of Medicine, King Saud University and King Saud University Medical City, Riyadh, Saudi Arabia; ^3^College of Medicine, King Saud University, Riyadh, Saudi Arabia

## Abstract

**Background:**

The clock drawing test (CDT) is frequently used to detect changes in cognition. Multiple scales of varying length have been published to assess performance. The aim of this study is to compare the CDT performance measured by three scales among a sample of nondemented patients on renal dialysis and identify the variables that affect performance. *Methodology*. This is a cross-sectional study performed at the dialysis unit at King Saud University Medical City. Eighty-nine dialysis patients performed the CDT. The CDT was scored by the methods of Rouleau et al. (RCS 10-point), Babins et al. (BCS 18-point), and the MoCA (MCS 3-point). Regression models were used to determine influencing demographic and dialysis variables. Scores were then correlated, and a combined factor analysis of scale components was done.

**Results:**

Females represented 44.6%, the mean (SD) age was 49.99 (15.49) years, and education duration was 10.29 (5.5) years. Dialysis vintage was 55.81 (62.91) months. The scores for the MCS, RCS, and BCS were 2.18 (1.08), 6.67 (3.07), and 11.8 (5.5), respectively, with significant correlation (*P* < 0.0001). In all scales, increasing age was associated with a lower score (each *P* < 0.0001). The scores increased with increasing education (each *P* < 0.0001). Diabetics had a lower score on both the BCS and MCS by 2.56 (SE 1.2) (*P* = 0.035) and 0.71 (*P* = 0.003) points, respectively. However, only age and years of education were significant in the multivariable analysis. In factor analysis, two shared factors appeared between the three scales: hand and number placement and the clock face.

**Conclusion:**

Age and education influence the performance on the CDT, and factors diverged into executive and visuospatial components. The MCS is likely to yield useful information but should be interpreted as part of the MoCA.

## 1. Introduction

The clock drawing test (CDT) is a classical test of cognition. Its earliest use was with patients suffering from parietal lobe lesions [1]. This test has remained popular over the years owing to its ability to assess executive function, visuospatial abilities, and short-term memory, giving it wide applicability to different disorders of cognition [2–6]. While the CDT is likely more useful in detecting dementia than mild cognitive impairment (MCI) [7, 8], its little influence from culture [9] and good test-retest reliability [10] make it a useful assessment tool [11] that correlates with other neuropsychological tests, such as the Mini-Mental State Exam (MMSE) [12], the dementia rating scale [13], the frontal assessment battery (FAB), and other measures of visuospatial and semantic functions [14]. The test has also been found to be complimentary to the MMSE in better detecting executive impairments [15]. The CDT is valuable in detecting impairments from various disorders, such as Alzheimer's disease [13], vascular cognitive impairment [4], Huntington's disease [3], and Parkinson's disease [5, 6].

Patients with end-stage renal disease (ESRD) have an increased risk of developing cognitive impairment. Severe dementia occurs in at least a third of ESRD patients [16]. Executive dysfunction is the most likely cognitive domain to be involved [17], while age and education are major influencers on tests of cognitive assessment in this population [17]. Considering that the CDT can be implemented by medical staff of various backgrounds [18], it can be used to easily screen ESRD patients for cognitive changes.

The numerous scoring scales for the CDT (which have varying levels of detail) raise the question about which one is the best to use in a busy clinical setting. One easy-to-use and highly familiar measure is the Montreal Cognitive Assessment (MoCA) tool [19], which simply grades the CDT with a score ranging from 0 to 3. There are more detailed scales, such as those by Rouleau et al. [3] and Babins et al. [20], which score the CDT on 10-point and 18-point scales, respectively. However, it is still not entirely clear how the simple method described in the MoCA corresponds with other more detailed scales, particularly in ESRD patients on dialysis. Therefore, the first aim of this study was to compare CDT results measured by three scoring systems among a sample of non-dementia-diagnosed ESRD patients on dialysis and identify which patient demographic, dialysis, or comorbidity variables would significantly influence their performance. The second aim of the study was to use an exploratory factor analysis to determine whether the short clock scale from the MoCA, with limited subcategories, would agree with the detailed subcomponents of two detailed clock scoring scales.

## 2. Methods

### 2.1. Participants and Setting

This is a quantitative, observational, cross-sectional study performed at the dialysis unit at King Saud University Medical City (KSUMC). Data collection was conducted between November 2018 and April 2019. A total of 89 patients with ESRD, over 18 years of age, who were on dialysis for at least 90 days, were included to eliminate any possibility of uremic effects. We excluded all illiterate patients, those diagnosed with a dementia disorder, or those with language or speech disorders. We also excluded patients who had active psychiatric disorders or were taking any substances that could affect the central nervous system (CNS), such as antipsychotic medications, sedatives, or opiates. Those with a history of brain injury, untreated metabolic disorders (e.g., thyroid disease and B12 deficiency), demyelinating disease, CNS vasculitis, or symptomatic hemorrhagic or ischemic strokes were also excluded. Demographic information, including age, gender, and years of education, was collected. Additionally, disease-related information, including the type of dialysis (peritoneal or hemodialysis), dialysis vintage, and the presence of diabetes and hypertension, was obtained. This study was approved by the internal review board at the College of Medicine, King Saud University, Riyadh, Saudi Arabia.

### 2.2. Tools

The MoCA is a brief screening tool used to diagnose early dementia and MCI by evaluating a range of cognitive domains: memory, language, naming, visuospatial, attention, and executive functions [19]. The CDT score of the MoCA (MCS) ranges from 0 to 3. The scale by Rouleau et al. (RCS) [3] assesses three components of the CDT, which is similar to the MoCA, except with a range from 0 to 10 points; scores ≤ 7 usually correspond to significant cognitive impairment [3, 7, 21]. The scale by Babins et al. (BCS) [20] is an 18-point system divided into subcategories to increase the sensitivity of scoring for earlier detection of impairments: scores below 13 indicate the development of dementia [20]. Each of the aforementioned scales has subcomponents for different aspects of clock drawing. The RCS has a clock face scale (RCSf) of 2 points, a number scale (RCSn) of 4 points, and a clock hand scale (RCSh) of 4 points. The BCS has a contour scale (BCSc) of 2 points, a center scale (BCScen) of 2 points, a number scale (BCSn) of 6 points, a clock hand timing scale (BCSht) of 3 points, a clock hand construction (BCShc) of 3 points, and a gestalt component (BCS) of 2 points. The scale used on the MoCA test gives a score of 0 or 1 to either the hands (mh), circle (Mc), or numbers (Mn). We chose these three scales (MCS, RCS, and BCS) because they had similar subcategories of clock circle, clock numbers, and clock hands. These predetermined components aid the analysis of similar subcomponent group together. The three scales were all quantitative measures that were easy to learn and did not rely on predrawn circles.

### 2.3. Procedure

Five senior medical students underwent training before using the scales under the supervision of a neurologist specialized in cognitive disorders. Prior to the scoring of the clocks, a total of 49 clock drawings obtained from other sources demonstrating various levels of performance were used to practice scoring and measure the reliability of the raters on each of the three scales. Each rater was assigned a specific day to assess patients receiving dialysis in order to randomly distribute participants among assessors. The participants in the current study were verbally instructed to draw a clock with a pen on a blank piece of white A4 paper and indicate the time with the two hands at “10 past 11.” The instructions were repeated if requested by the participant at any point during the task. All participants were native Arabic speakers; therefore, the instructions were provided in the Arabic language. For the demonstration of the time, the Arabic instructions would literally translate to “eleven and ten,” thereby still preserving the requisite for the patient to mentally convert the “ten” to the “two” on the clock face. The clocks drawn were later scored by the raters using the MCS. After at least a month had passed, the same clocks were scored using both the BCS and RCS in random order without knowledge of the MCS score.

### 2.4. Analysis

The sample size was estimated based on the differences obtained from previous studies of clock drawings [3, 13, 20] that compared cognitively normal with cognitively impaired individuals. We expected our sample to contain a range of cognitively normal people to those with mild cognitive impairment. Thus, we based the sample size on the ability of the scales to make this discrimination with a 0.05 alpha and 0.9 power, which was about 55 patients. Descriptive statistics was used to assess means, medians, and proportions for demographic variables. Spearman correlations were used to test the significance of the correlations between MCS, BCS, and RCS. Age and years of education were also categorized to determine group effects. To assess the hypothesis on whether predictions can be made for the scales, multivariable regression was used to assess the influence of each independent variable. Only those variables found to be significant in univariate regression were entered into the multivariable model. A *P* value of <0.05 was considered statistically significant. Exploratory factor analysis (EFA) was used to assess the hypothesis on whether there are any latent constructs or if the variables loaded similarly. To determine where the MCS components loaded in relation to the two detailed scales, all components of the three scales were included in a single EFA. Relevant factors were those with eigenvalues above one. Bartlett's test of sphericity and the Kaiser-Meyer-Olkin (KMO) measure were used for determining intercorrelation and sampling adequacy, respectively. Stata 15 software was used for analysis.

## 3. Results

**Figure 1:**
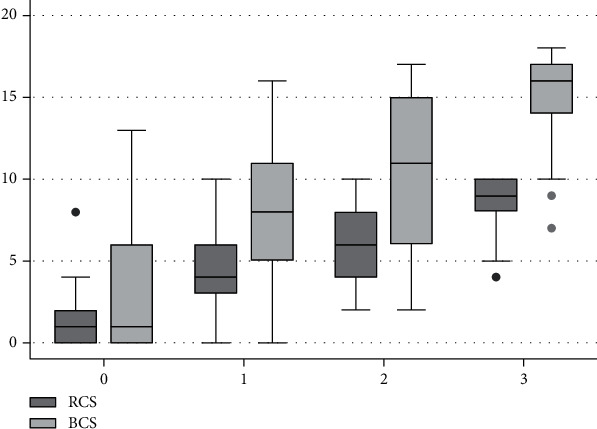
Distribution of the mean scores of the RCS (0-10) and BCS (0-18) (*y*-axis) according to the performance on the MCS (0-3) (*x*-axis) demonstrating the relation of scores obtained from patients on all the three scales.

The vast majority of participants were right-handed (78 or 96.3%), with the remaining being left-handed. The mean age was 49.99 with a standard deviation (SD) of 15.49 years. The mean years of education was 10.29 with a SD of 5.5. The mean (SD) duration of dialysis (dialysis vintage) was 55.81 (62.91) months (median of 31 months), 63.2 (71.44) months for hemodialysis patients, and 41.04 (37.88) months for peritoneal dialysis patients. The mean (SD) and median with interquartile range (IQR) scores for the MCS, RCS, and BCS (in addition to the performance on each task according to demographic features and comorbidity) are demonstrated in Table 1 and Figure 1.

Figure 1 shows the distribution and relation of scores for patients on all the three scales. A correlation analysis showed that the MCS correlated to the same degree with the BCS and RCS, both at 0.7 (*P* < 0.0001). The RCS and BCS correlated even stronger at 0.88 (*P* < 0.0001). The intraclass correlation by the five raters was excellent at 0.88, 0.93, and 0.93 for MCS, RCS, and BCS, respectively (each *P* < 0.0001). Patients who obtained a score of >7 on the RCS and ≥13 on the BCS had corresponding mean (SD) scores on the MCS equal to 2.77 (0.59) and 2.77 (0.58), respectively.

Univariate regression was performed with each of the three clock scales as a dependent variable. Gender was not a significant variable for BCS, RCS, or MCS (*P* = 0.51, *P* = 0.57, and *P* = 0.34, respectively). Similarly, the duration of dialysis was not significant for any of the three scales either (*P* = 0.21, *P* = 0.23, and *P* = 0.183, respectively). The dialysis type was also not significant (*P* = 0.14, *P* = 0.06, and *P* = 0.19, respectively). The RCS showed no significant association with diabetes (*P* = 0.08), while there was a significant relationship with the BCS and MCS (Table 2). Multivariable regression (Table 2) analyses were employed using only the significant variables in the univariate analysis as independent variables and the scores of the BCS, MCS, and RCS as dependent variables. In all regression models, only age and years of education were significantly associated with the dependent variable. Tests of significance could not be carried out on participants with hypertension due to the small numbers of nonhypertensive participants.

The EFA showed that the components from each of the three scales loaded on two factors with eigenvalues of 7.36 and 1.28, which, respectively, explained 61.32% and 10.68% of the variance. Bartlett's test of sphericity was <0.0001, and the Kaiser-Meyer-Olkin (KMO) was 0.896, indicating adequate sampling. After oblique rotation, factor loadings with values above 0.4 were retained (Table 3).

## 4. Discussion

This study demonstrates a relationship between three different clock drawing scales that varied widely in their scoring range in a group of dialysis patients, and significant variables that influenced performance were identified. All three scales correlated well with each other and were quickly learned with good reliability among raters.

All scales showed significant differences depending on age and education, with lower scores in older individuals and in those with less education. This is not only consistent with the usual variations generally seen in cognitive abilities [22] but also with previous findings that demonstrated frequent cognitive impairment in elderly individuals with ESRD [23], particularly in the CDT. These two factors also appear to be the main influencers on the CDT performance, whereas other variables (such as dialysis vintage and types of dialysis methods) showed no effect. This has similarly been demonstrated in other studies of ESRD patients [9, 17] in which age and education were the main factors influencing performance on cognitive assessment scales [24]. There appeared to be initially a suggestion of an effect from diabetes; however, this was not supported in the multivariable models. This requires further exploration with larger numbers of patients, particularly since diabetes is well known to be associated with cognitive disorders (including vascular dementia and Alzheimer's disease [25]). An additional important observation in this study is the presence of participants with low performance scores on the CDT who were not previously recognized to have any cognitive limitations. This finding is similar to previous studies that describe unexpected cognitive impairment in ESRD patients using various neuropsychological tests, including the CDT [24, 26], in patients not previously known to have dementia or cognitive impairment.

The small-ranged scale used on the MoCA significantly varied among patients. While a zero on the MCS correlated with very low points on the other two longer scales, other patients might score just below the proposed cut-off yet have a full score on the MCS (Figure 1). Interestingly, the mean MCS score for clocks with scores above the cut-off of each of the longer scales corresponded to 2.77 on the MCS. Therefore, a loss of even one point on the MCS would trigger a need to investigate further. On the other hand, a full score might not rule out minor impairments in a few patients. These considerations are important if the MCS is to be used as a standalone measurement; however, it is generally scored as part of the entire MoCA and additional findings on the assessment help identify further impairments and corroborate the significance of any clock abnormalities. Consistent with our objective of identifying agreement between the subcomponents of the three scales, we found that all variables did not load equally, but, rather, the three scales shared underlying structures. Numbering and hand placement components of the MCS shared the same factor as did the numbering and hand components of the two detailed scales on the EFA. Similarly, the circle and clock face components appeared to have a separate factor that explained a smaller amount of the variance. The fact that executive impairment is common in ESRD patients [17] explains why this two-factor finding emerged. Hand and number errors usually start out as impairments of execution, whereas an individual with more severe impairments affecting cognitive elements of knowledge, conception, or visuospatial representation of clocks is likely to have difficulties drawing the clock from the start: namely, the circle [3, 27]. While the two long scales have predetermined usefulness [13, 18, 20], the information obtained from the MCS is likely to be similarly useful in identifying errors prior to determining whether more detailed cognitive assessments would be needed, making it a quick and useful screening tool. It is conceivable that this applicability will expand beyond ESRD patients to other conditions for which executive impairments are common. An example would be Parkinson's disease, in which the CDT has been found to be more sensitive than the MMSE in identifying cognitively impaired patients [5].

Among the limitations to consider in this study is that hypertension was a prevalent diagnosis, and few patients were nonhypertensive. This limited our ability to determine the effect of blood pressure on performance. Also, while not all our patients were diagnosed with dementia, patients with mild cognitive impairment were not identified prior to testing. In fact, it was likely beneficial to not exclude mild cognitive impairment to allow for a broader spectrum of patients in order to better test the scales' correlations. Future studies that examine further the sensitivity and specificity of the MCS would be warranted, given its narrow scoring range.

In summary, age and education significantly influence performance on the CDT as demonstrated on all three scales involving patients with ESRD. The short scale for assessing performance on the CDT is likely to yield useful information and is in agreement with the longer scales. While it is structured similar to the more detailed scales, it still does not have enough depth to uncover the details of the impairment, especially if not used in conjunction with the entire MoCA; however, it is adequate to trigger suspicion of a significant cognitive deficit in ESRD patients given the frequency of executive dysfunction and impairments on the CDT in this population.

## Figures and Tables

**Table 1:** Baseline patient data and their performance on each of the scales. SD: standard deviation; IQR: interquartile range.

		*N* (%)	MCS	RCS	BCS
			Mean (SD) Median (IQR)		
Entire group		83	2.18 (1.08) 3 (1)	6.67 (3.07) 8 (5)	11.8 (5.5) 14 (9)
Gender	Male	46 (55.42)	2.28 (0.98) 3 (1)	6.85 (2.72) 8 (4)	12.15 (5.13) 14 (9)
	Female	37 (44.58)	2.05 (1.2) 3 (1)	6.46 (3.49) 8 (6)	11.35 (6) 14 (8)
Age group	<40	21 (25.3)	2.62 (0.8) 3 (0)	8.29 (2.26) 9 (2)	13.95 (4.13) 15 (2)
	40-59	37 (44.58)	2.43 (0.8) 3 (1)	7.22 (2.62) 8 (3)	12.97 (4.84) 15 (6)
	>59	25 (30.12)	1.44 (1.29) 1 (3)	4.52 (3.19) 4 (6)	8.24 (5.88) 8 (8)
Education group	<9 years	22 (28.57)	1.18 (1.3) 1 (3)	4.27 (3.55) 4 (7)	7.55 (6.43) 7 (15)
	9-12 years	31 (40.26)	2.58 (0.62) 3 (1)	7.55 (2.17) 8 (3)	13.58 (3.52) 15 (4)
	>12 years	24 (31.17)	2.67 (0.76) 3 (0)	7.83 (2.43) 8 (2)	13.67 (4.63) 15 (3)
Diabetes	Yes	35 (42.17)	1.77 (1.3) 2 (3)	5.97 (3.35) 7 (5)	10.31 (5.9) 13 (10)
	No	48 (57.83)	2.48 (0.82) 3 (1)	7.19 (2.77) 8 (3)	12.88 (4.97) 15 (5)
Hypertension	Yes	71 (85.54)	2.15 (1.1) 3 (2)	6.45 (3.09) 8 (5)	11.37 (5.6) 13 (9)
	No	12 (14.46)	2.33 (0.98) 3 (1)	8 (2.7) 9 (2)	14.33 (4.21) 16 (2)
Dialysis type	Hemodialysis	56 (67.47)	2.07 (1.13) 2.5 (2)	6.23 (3.26) 7.5 (5)	11.18 (5.81) 13 (9)
	Peritoneal dialysis	27 (32.53)	2.41 (0.97) 3 (1)	7.59 (2.45) 8 (2)	13.07 (4.62) 15 (3)

**Table 2:** Multivariable regression with variables that were significant in the univariate analysis for the BCS, RCS, and MCS.

		Univariate			Multivariable		
		Coefficient	SE	*P* value	Coefficient	SE	*P* value
BCS	Age	-0.15	0.04	<0.0001	-0.1	0.04	0.01
	Years of education	0.45	0.1	<0.0001	0.34	0.11	0.003
	Diabetes	-2.56	1.2	0.04	-0.34	1.2	0.77
RCS	Age	-0.09	0.02	<0.0001	-0.07	0.02	0.001
	Years of education	0.26	0.06	<0.0001	0.19	0.06	0.001
MCS	Age	-0.03	-0.01	<0.0001	-0.02	0.01	0.02
	Years of education	0.11	0.02	<0.0001	0.08	0.02	<0.0001
	Diabetes	-0.71	0.23	0.003	-0.22	0.22	0.32

**Table 3:** Factor analysis of subcomponents of all the three scales.

Clock scale component	Factor 1	Factor 2
Numbers (BCS)	0.94	
Time (BCS)	0.82	
Construct of hands (BCS)	0.68	
Gestalt (BCS)	0.92	
Presence and placement of hands (RCS)	0.79	
Numbers (MCS)	0.82	
Hands (MCS)	0.7	
Contour integrity of the clock face (BCS)		0.96
Center (BCS)		0.43
Integrity of the clock face (RCS)		0.97
Contour (MCS)		0.65

## Data Availability

The datasets used and/or analyzed during the current study are available from the corresponding author on reasonable request.

## Abbreviations

BCS: Babins clock scale
CDT: Clock drawing test
ESRD: End-stage renal disease
EFA: Exploratory factor analysis
IQR: Interquartile range
MoCA: Montreal Cognitive Assessment
MCS: MoCA clock scale
RCS: Rouleau clock scale
SD: Standard deviation.
